# A rare case of Fanconi anemia with Mitomycin C sensitivity: A pediatrics case report

**DOI:** 10.1002/ccr3.8711

**Published:** 2024-03-26

**Authors:** Vraj Bhatt, Sunidhi Rohatgi, Mansi Singh

**Affiliations:** ^1^ Department of Medicine Baroda Medical College, S.S.G Hospital Vadodara Gujarat India; ^2^ Department of Medicine Bogomolets National Medical University Kyiv Ukraine

**Keywords:** chromosome breakage analysis, Fanconi anemia, Mitomycin C sensitivity, radial hypoplasia, rare genetic disorder, stem cell transplant

## Abstract

**Key Clinical Message:**

Fanconi anemia with Mitomycin C sensitivity is a rare, complex hematological condition. Our case study emphasizes the significance of early diagnosis, appropriate genetic testing, and cautious use of chemotherapeutic agents.

**Abstract:**

Fanconi anemia (FA) is a rare genetic disorder characterized by bone marrow failure, congenital anomalies, and predisposition to cancer. Here, we present the case of a 6‐year‐old boy with a known diagnosis of Fanconi anemia who exhibited sensitivity to Mitomycin C. The patient had a history of recurrent blood transfusions due to anemia and was referred to our institution following worsening symptoms, including pallor, swelling in limbs, and respiratory distress. Physical examination revealed characteristic features of FA such as mesomelia, low‐set ears, hyperpigmented macules, microcephaly, micropthalmos, and thumb hypoplasia. Imaging studies demonstrated bilateral radial hypoplasia and congenital agenesis of the left kidney. Laboratory investigations revealed pancytopenia, aberrant liver function tests, and elevated inflammatory markers. Importantly, the patient exhibited sensitivity to Mitomycin C, highlighting the necessity for caution in selecting chemotherapeutic agents in FA patients. This case underscores the importance of early recognition, comprehensive evaluation, and tailored management strategies of patients with Fanconi anemia to optimize outcomes and minimize complications.

## 1 INTRODUCTION

Fanconi anemia (FA) represents a rare hereditary condition marked by bone marrow failure, congenital malformations, and an elevated susceptibility to cancer. It predominantly affects pediatric populations and is typified by various physical anomalies evident from birth. Individuals with FA commonly display symptoms including anemia, thrombocytopenia, leukopenia, and heightened sensitivity to agents causing DNA damage.

Fanconi anemia is a rare type of anemia with an incidence of 1 in 3,50,000 in India.^1^ More prevalence is seen in the South African people. The Ashkenazi community is more likely to suffer from this illness. Compared to black South Africans, who have a carrier frequency of one in 40,000, Ashkenazi Jews have a disease carrier frequency of one in 89.^2^ It is the most common inherited form of aplastic anemia. It is a genetically heterogenous autosomal recessive condition^3^ (most common form). The disease is evident due to chromosomal instability that affects the proteins involved in DNA repair and regulation of cell cycle.

## 2 CASE HISTORY

A referred case of a 6‐year‐old boy who was known to have Fanconi anemia was in a tertiary hospital on June 11, 2023. Six months before the admission, the patient went to a private hospital in Vadodara, India, and made three visits (in January, March, and May) in a span of 6 months. Every time, he was administered 2 units of 100 mL blood, and the hemoglobin came back to normal range after infusions. The private hospital then referred the patient to a tertiary hospital. Initially, for 5 years, the patient was asymptomatic, and earlier this year, the patient developed intensifying dry cough, vomiting, and abdominal pain, more frequently at night, and so the patient was transported to a private hospital. The patient's parents and two siblings remain unaffected. The reporting team checked for hematological profile to evaluate the amount of blood to be transfused.

**FIGURE 1:**
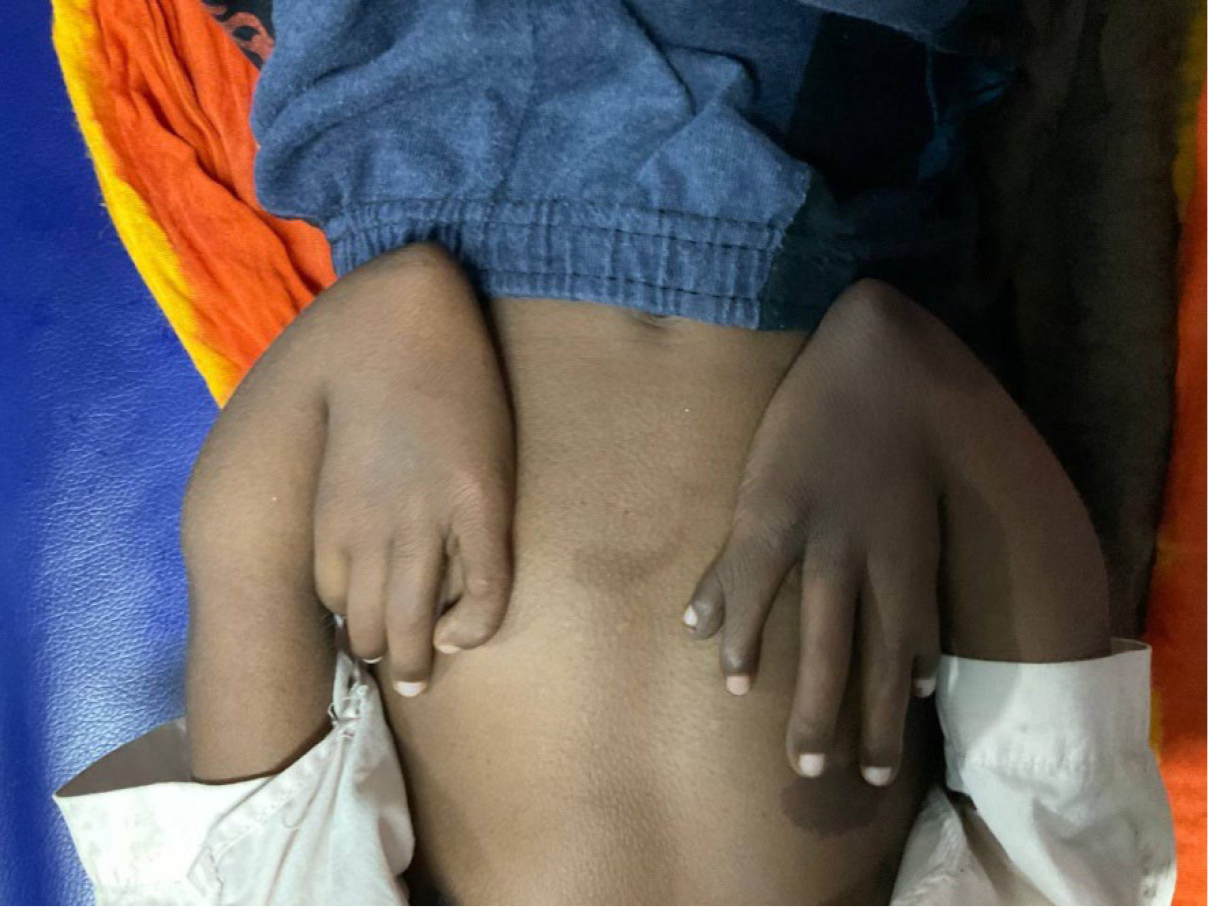
Hand anomalies and hyperpigmented macules.

On general examination, the patient had considerable pallor on the tongue, mucosal linings, and nails. Physical examinations showed mesomelia, low‐set ears, multiple hyperpigmented macules, microcephaly, micropthalmos, and thumb hypoplasia in both hands (Figure 1). The testicles were bilaterally undescended, and the abdomen was soft and non‐tender. All developmental milestones have been attained by the patient. He has a history of PCV (Packed Cell Volume) transfusions for the last 6 months.

## 3 METHODS

Investigations included differential WBC count and full blood count. The biochemical investigation (Table 1) revealed a normal urea level, normal thyroid function, and elevated C‐reactive protein in addition to an aberrant liver profile (with elevated S.AST and S.ALT) and normal electrolytes. Also, the hematological profile supported the patient's pancytopenia as the patient has decreased Red blood cell count, platelet count, and total WBC count, manifesting as episodes of epistaxis and bacterial infections in last 6 months. The patient's Hemoglobin was below normal levels as well. The red cell distribution width was more than normal levels due to bone marrow stress erythropoiesis, which leads to premature release of immature cells into the bloodstream.^4^ Urinalysis showed increased levels of glucose and amino acids and low levels of Bicarbonate.

**TABLE 1:** Biochemical investigation.

Parameter	Patient value	Biological reference range
HAEMOGRAM		
Hemoglobin (g/dL)	**6.9**	13–17
Red blood cell count (/L)	**2.87 million**	4–5.5 million
Hematocrit (%)	**21.8**	34–40
RDW	**20%**	11.5%–15%
Total WBC Count (/cmm)	**4930**	5000–15,000
Differential WBC Count (/cmm)	**2600**	3000–7000
Platelet count (/micro liter)	**86,000**	150,000–450,000
BIOCHEMISTRY		
S.ALT (IU/L)	**71.7**	10–50
S.AST (IU/L)	**66.6**	10–40
S.ALP (IU/L)	324	50–450
S. Sodium (mEq/L)	137	135–145
S. Potassium (mEq/L)	4.2	3.5–5.5
S. Urea (mEq/L)	19.2	10–20
S. Creatinine (mEq/L)	0.9	0.7–1.3
S. Ferritin (ng/ml)	**1264**	20–250
S. CRP (mg/L)	**17.20**	0–6
S. TSH (uIU/ml)	2.38	0.38–5.3
S. T3 (ng/ml)	1.49	0.89–1.7
S. T4 (μg/dL)	6.160	6.03–11.5

*Note*: The bold values highlight the abnormal range.

**FIGURE 2:**
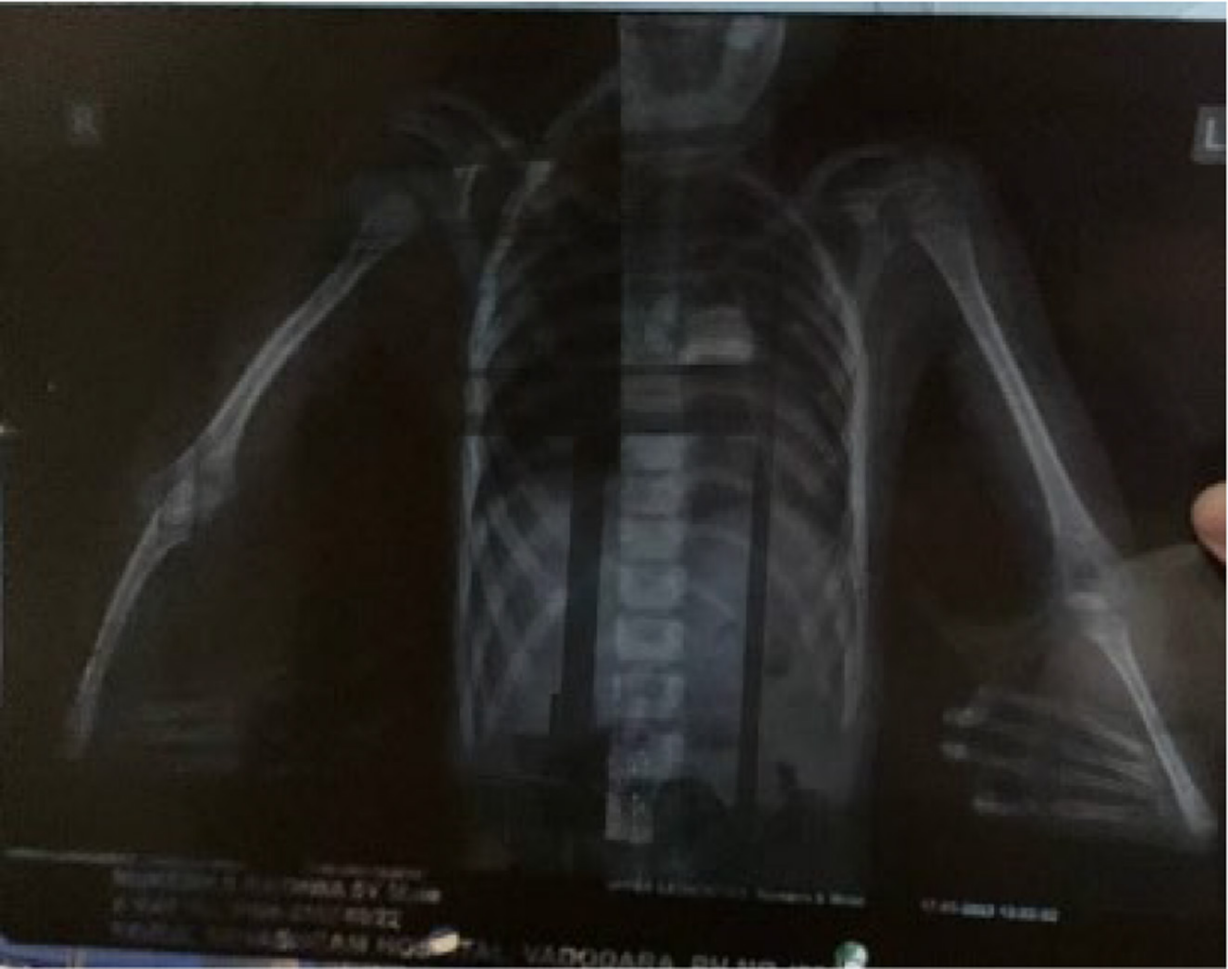
Plain chest X‐ray showing bilateral radial hypoplasia.

**FIGURE 3:**
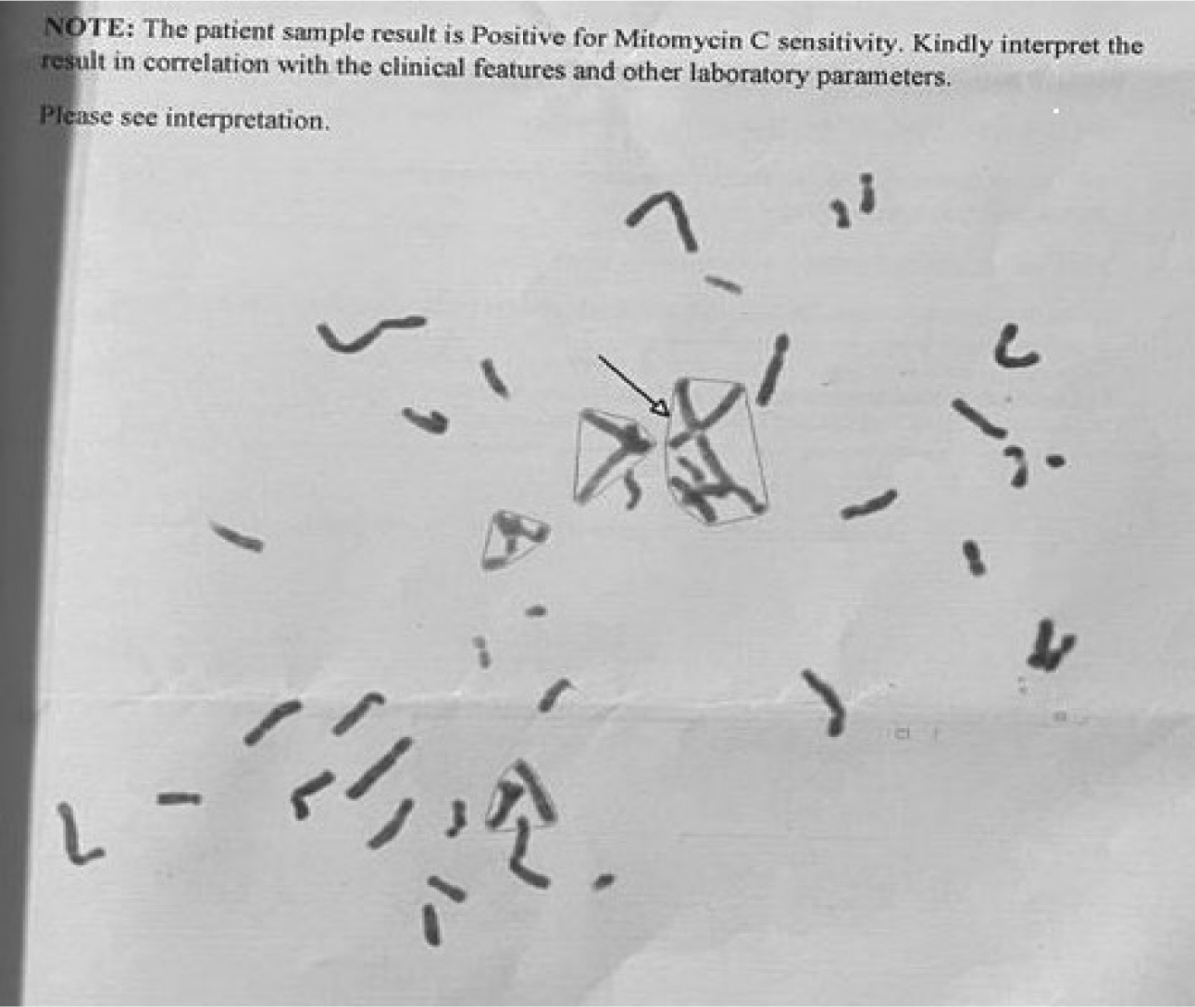
Chromosomal breakage test using Mitomycin C.

A USG was performed, which revealed a right kidney with a dilated calyceal system and the absence of the left kidney in the left renal or iliac fossa. This is due to congenital agenesis of left kidney. The X‐ray revealed bilateral radial hypoplasia (Figure 2), which is a typical feature of Fanconi anemia. Chromosomal breakage test was performed using Mitomycin C in a peripheral lymphocyte culture (Figure 3). The Mitomycin C sensitivity was calculated using Triradials/Quadriradials formula (Table 2).

**TABLE 2:** Calculating Mitomycin C sensitivity.

Mitomycin C sensitivity calculation: = Percentage of cells with Triradials/Quadriradials +1.6 times the total number of Triradials/Quadriradials = 98 + (1.6 * 96) =98 + 153.6 =251.6 (Normal 10 or less)
Conclusion: Sample sensitive to Mitomycin C

## 4 CONCLUSION AND OUTCOME

By the clinical findings of mesomelia, thumb hypoplasia, renal agenesis, and investigations like Complete blood count and Mitomycin C sensitivity test, the patient was diagnosed with Fanconi anemia.

The patient was managed with 3 PCV transfusions, the amount is calculated by multiplying unit – 10 mL/kg. Hence, 100 mL of blood per pint. Also, I.V. fluids, paracetamol, folic acid syrup (5 mL) and multivitamin B complex syrup (5 mL).

He was started on steroid Cap Danazol 5 mg/kg/day.

Folic acid supplementation is started to stimulate the erythropoesis with the goal of increasing the amount of oxygen carried by RBC.

**Table:** 

Follow up	1st	2nd
Date	15/08/23	17/09/23
Any complaints	Fever	Weakness
Presented Hb	3.1 mg/dL	3.2 mg/dL
Post treatment Hb	9.32 mg/dL	10.3 mg/dL
Duration of stay	2 days	3 days
PCV pints administered	2 units of 100 mL	2 units of 100 mL
Any medicines given	Folic acid tablets	Folic acid tablets and Danazol
Any new findings	None	Raised serum ferritin (infection, liver disease)

Learning points:

1. There should be more awareness and knowledge to common people about this disease. This should also include genetic counseling that is to be given to the parents about the possibility of transfer of genes to the next generation.
2. The definite treatment of bone marrow transplant is not economically feasible for lower economic strata. Hence, to give feasible and subsidiary treatment, more research needs to be conducted.
3. It helps us to differentiate Fanconi anemia from all other causes of severe anemia and pancytopenia and to differentiate it from Ataxia telangiectasia, Bloom syndrome, and paroxysomal nocturnal hemoglobinuria.

## 5 DISCUSSION

People of various races have been diagnosed with Fanconi anemia. The heterozygote frequency is higher in South African Afrikaners due to founder effects, though. One in every 3,60,000 people in India has the condition. Patients with a median age of 7 years are evidently seen having this disease. Also, a sex ratio of 1:1 is seen among patients suffering from this disease.^5^ It has an autosomal recessive etiology, meaning that patients with it can be homozygous or double heterozygous. To date, 23 genes (FANCA, FANCB, FANCC, FANCD1/BRCA2, FANCD2, FANCE, FANCF, FANCG, FANCI, FANCJ/BRIP1, FANCL, FANCM, FANCN/PALB2, FANCO/RAD51C, FANCP/SLX4, FANCQ/ERCC4, FANCR/RAD51, FANCS/BRCA1, FANCT/UBE2T, FANCU/XRCC2, FANCV/REV7, FANCW/RFWD3, and FANCY/FAP100) have been discovered to play a role in the FA pathway.^6^ FANCA mutations account for 60%–65% of all FA patients globally and are the most common of them. These genes produce proteins that are involved in the FA/BRCA repair pathway, which locates covalently bound damage to the two DNA strands and performs its repair through homologous monoubiquitination and recombination. The result of their absence is interstate crosslinks (ICL). At a chromosome breakage study of chromosomes at the metaphase stage, this results in DNA breaking and nonhomologous end‐joining of free ends, which are visible and countable. As a result, affected individuals have chromosomal instability, genome damage, and a significant chance of developing cancer and congenital abnormalities.^5^ Clinically, the patient's entire body is impacted. Low birth weight, short pre‐ and postnatal height, and microcephaly are among the physical anomalies identified. When micrognathia, triangular face, and head and neck micropthalmia are present, significant clinical suspicion should be aroused. Persistent aortic duct coarctation and situs inversus totalis have both been observed in CVS. The most indicative but not the most sensitive limb examination reveals mesomelia, radial hypoplasia, and hypothenar eminence hypoplasia. We can appreciate low‐set ears and hyperpigmented patches of the color of coffee, which are not limited to ears and can spread to the back, chest, and abdomen of the patient. The patient can also have conductive hearing loss. Agenesis and horseshoe kidneys are examples of genitourinary system anomalies, as are misplaced testicles or ovaries. The patient's small height is a result of insulin resistance, hypothyroidism, and growth hormone deficit in the endocrine system. Last but not least, pancytopenia, acute myeloid leukemia, bone marrow failure, and myelodysplastic failure are all seen in hematology.

Echocardiogram is used to look for any structural cardiac anomalies. Bony flaws should also be checked with an X‐ray. The absence of the left kidney was also confirmed by USG. The results of the laboratory tests point to aberrant liver function, hyponatremia, and elevated CRP levels, which are due to increased production of inflammatory mediators in response to an underlying infection or degenerative disorder.

We perform a chromosomal breakage test^5^ to determine the illness and its chromosome arrangement. If one is not sensitive to Mitomycin C^3^ and there is no clinical indication of Fanconi anemia, it is likely to be any other abnormality. But here, it is Mitomycin C sensitive (Table 2). However, skin fibroblast testing could be performed if clinical suspicion is present in order to rule out mosaicism. Additionally, gene mutation testing to rule out additional chromosomal breakage‐related diseases include Ataxia telangiectesia, Bloom syndrome, Roberts syndrome, DNA Ligase 4 syndrome, and Warsaw breakage syndrome. De Novo myelodysplastic syndrome, drug‐ or infection‐induced pancytopenia, and paroxysomal nocturnal hemoglobinuria are further differential diagnoses.

Additionally, the parents should receive genetic counseling and instruction regarding the syndrome's inheritance pattern. Symptom‐supportive care can be used to manage the sick patient. The best treatment is blood transfusions, which include transfusions of packed RBCs and platelets.^7^ Folic acid tablets and steroids like Danazol for children could be administered. For specific treatment of the deformities, surgical methods, androgen therapy, gene therapy, bone marrow transplant, and Hematopoietic stem cell transplant (best curative) are used. The survival rate after transplant was 66% in the case of identical sibling transplants.^8^

The parents should be instructed by the physician to bring the child for monthly follow‐up appointments, during which hemoglobin and complete blood count investigations will be conducted. The volume of blood transfusions should be adjusted accordingly. Additionally, the patient's fever and other laboratory serum inflammatory markers, such as CRP and ferritin, need to be monitored for any elevation indicative of infection, progression towards cancer, or the development of any degenerative condition.

As the disease involves a germline mutation, cancer screening is also conducted every 6 months. Squamous cell cancers of the head and neck area, particularly of the tongue and oral mucosa, followed by the esophagus, anogenital, and urinary system, are observed in this disease. Early detection of cancer is crucial for early treatment and is deemed vital for improving overall survival in individuals with FA.

## AUTHOR CONTRIBUTIONS

**Vraj Bhatt:** Data curation; investigation; writing – original draft. **Sunidhi Rohatgi:** Methodology; resources; writing – original draft. **Mansi Singh:** Conceptualization; formal analysis; writing – review and editing.

## FUNDING INFORMATION

No funding to disclose.

## CONFLICT OF INTEREST STATEMENT

None.

## CONSENT

Written informed consent was obtained from the patient to publish this report in accordance with the journal's patient consent policy.

## Data Availability

The datasets analyzed during the current study are available from the corresponding author upon reasonable request. Additionally, comprehensive literature sources used for the literature review are cited appropriately within the manuscript.
